# Archaeal Clusters of Orthologous Genes (arCOGs): An Update and Application for Analysis of Shared Features between Thermococcales, Methanococcales, and Methanobacteriales

**DOI:** 10.3390/life5010818

**Published:** 2015-03-10

**Authors:** Kira S. Makarova, Yuri I. Wolf, Eugene V. Koonin

**Affiliations:** National Center for Biotechnology Information, NLM, National Institutes of Health, Bethesda, MD 20894, USA; E-Mails: wolf@ncbi.nlm.nih.gov (Y.I.W.); koonin@ncbi.nlm.nih.gov (E.V.K.)

**Keywords:** arCOGs, genome annotation, phylogenomics, Thermococci, methanogens

## Abstract

With the continuously accelerating genome sequencing from diverse groups of archaea and bacteria, accurate identification of gene orthology and availability of readily expandable clusters of orthologous genes are essential for the functional annotation of new genomes. We report an update of the collection of archaeal Clusters of Orthologous Genes (arCOGs) to cover, on average, 91% of the protein-coding genes in 168 archaeal genomes. The new arCOGs were constructed using refined algorithms for orthology identification combined with extensive manual curation, including incorporation of the results of several completed and ongoing research projects in archaeal genomics. A new level of classification is introduced, superclusters that unit two or more arCOGs and more completely reflect gene family evolution than individual, disconnected arCOGs. Assessment of the current archaeal genome annotation in public databases indicates that consistent use of arCOGs can significantly improve the annotation quality. In addition to their utility for genome annotation, arCOGs also are a platform for phylogenomic analysis. We explore this aspect of arCOGs by performing a phylogenomic study of the Thermococci that are traditionally viewed as the basal branch of the Euryarchaeota. The results of phylogenomic analysis that involved both comparison of multiple phylogenetic trees and a search for putative derived shared characters by using phyletic patterns extracted from the arCOGs reveal a likely evolutionary relationship between the Thermococci, Methanococci, and Methanobacteria. The arCOGs are expected to be instrumental for a comprehensive phylogenomic study of the archaea.

## 1. Introduction

The quality of genome annotation and the utility of comparative genomic analysis critically depend on the accuracy of sequence comparison and reconstruction of gene and genome evolution. Evolutionary reconstruction is complicated by frequent events of gene duplication, gene loss and gain, typically, via horizontal gene transfer (HGT), gene fusions and fissions, and changing rates of gene evolution. Numerous genomic and metagenomic projects brought about an avalanche of genome sequences. Comparative analysis of these sequences increasingly relies on expert-curated databases of orthologous genes in which the common problems of automatic clustering and sequence comparison are systematically addressed. Otherwise, many errors are propagated any time when the most common sequence comparison approaches are used on the new set of genomes. Several sophisticated methods of automatic sequence clustering for the construction of orthologous gene groups have been proposed [1,2,3,4]. However, to attain accurate orthology identification and functional prediction, clusters produced by each of these approaches require continuous expert curation and additional methods such as phylogenetic analysis [5,6,7,8,9].

The database of archaeal Clusters of Orthologous Genes (arCOGs) was first developed in 2007 and initially covered 41 archaeal genomes [10]. Over the past years, a comprehensive update has been released, and methods for assigning arCOG membership have been refined in an attempt to achieve higher sensitivity and selectivity [11]. Importantly, manual curation of the arCOGs incorporated results of relevant research projects on archaeal genomics and evolution (e.g., [12,13,14,15,16,17,18,19,20,21,22]). The arCOGs database has become an important tool for archaeal genome analysis and annotation [19,23,24,25,26,27,28,29,30,31,32,33,34]. In particular, arCOGs have been included as the main resource for protein function annotation in archaeal genomes in the UCSC Archaeal Genome Browser [35].

Here we present a substantial update of the arCOGs that includes an extensive revision of the arCOG annotations based on comparison with the pfam, Conserved Domain Database (CDD), tigrfams, and recently updated COGs databases, literature searching, and data from several comparative genomic and phylogenomic studies. This effort resulted in the manual correction of both the composition and the annotation of many arCOGs. We also demonstrate how the arCOGs database can be efficiently used to address important evolutionary questions, by exploring shared features between the Thermococci and class I methanogenes (Methanococci, Methanobacteria, and *Methonopyrus*) [36]. The results of this analysis put into question the currently accepted position of the Thermococci in the archaeal phylogeny.

## 2. Materials and Methods

### 2.1. Genome Sequences and Basic Sequence Analysis

Genome sequences were downloaded from the NCBI FTP site [37]. New proteomes were assigned to the previous version of arCOGs using a RPS-BLAST search with a set of position-specific scoring matrices that were generated from the multiple alignments of the protein sequences of each arCOG [11]. Proteins that were not included in the existing arCOGs were clustered to create new arCOGs, as described previously [10]. Annotations of arCOGs that were linked to COGs were adopted when appropriate from recent updates in COG annotation [38]. A PSI-BLAST search [39] against the profiles available in the current version of the CDD (Conserved Domain Database) [40] was used to find the closest hits from arCOGs to pfam [41], conserved domain families [40], and tigrfams [42]. All annotations from these sources were compared with each other and the existing arCOG annotation and transferred to the respective arCOGs when appropriate. For the “uncharacterized” arCOGs remaining after the above approach, publicly available annotation of respective proteins has been examined and verified using PSI-BLAST and HHpred [43] programs and/or the literature search.

Transmembrane segments in protein sequences were predicted using the TMHMM v. 2.0c program with default parameters [44]. Signal peptides were predicted using the SignalP v. 4.1c program; the union of the three predictions (gram-negative, gram-positive, and eukaryotic models) was used [45]. Uncharacterized protein families for which two or more transmembrane segments were predicted were renamed to “Uncharacterized membrane protein”.

Multiple sequence alignments were constructed using a script combining MUSCLE [46] to align closely related sequences and MAFFT [47] to merge alignments. For phylogenetic reconstruction sites with a gap character fraction >0.5 and homogeneity <0.1 [48] were removed. The FastTree program [49] with WAG evolutionary model and discrete gamma model with 20 rate categories was used for phylogenetic tree reconstruction.

### 2.2. Genome Weighting

Estimates that involve aggregation of data from multiple genome sequences are often affected by sampling bias. In Archaea, some phyla (Korarchaeota) are represented by a single genome (*Candidatus* Korarchaeum cryptofilum OPF8), whereas the *Sulfolobus islandicus* species is represented by 10 genomes of different isolates. To mitigate this bias, we introduce relative genome weights.

The concept of relative genome weights is based on two intuitive notions: first, closely related genomes should contribute individually less to the total clade weight than their more distant relatives; second, the relative contribution of the clades should reflect the number of independent evolutionary events that occurred in the history of the clade. To the extent that the genome-level evolutionary scenario can be represented by a tree-like graph, the sum of branch lengths in a (sub-) tree naturally embodies both concepts.

More formally, consider a node in a rooted phylogenetic tree that has several descendant subtrees, each with the sum of its descendant branch lengths of *T_i_* and connected to the parent node by a branch of the length *L_i_*. If the descendant node is a leaf, it has no descendants, *i.e.*, *T_i_* = 0. If the total weight assigned to this node is set to *W*, then it is distributed between the descendant subtrees as *W_i_* = *W*(*L_i_* + *T_i_*)/Σ(*L_k_* + *T_k_*). Given a phylogenetic tree with branch lengths, the sums of branch lengths for each node can be easily computed iteratively in the leaf-to-root direction and the total tree weight can be iteratively distributed between clades and leaves in the root-to-leaf direction.

For the purpose of this work, we used an approximate phylogenetic tree reconstructed from concatenated alignments of ribosomal proteins that are universal among the archaea [12]. This tree was rooted between the common ancestor of Euryarchaeota and Nanoarchaeota and the common ancestor of the TACK superphylum (an assemblage of archaeal phyla that includes Taumarchaeota, Crenarchaeota, Korarchaeota, and Candidatus Caldiarchaeum subterreneum, only known the representative of the potential phylum Aigarchaeota) using a bacterial outgroup. This position of the archaeal root has been recently supported using a different set of markers that are shared between archaea and bacteria [50]. The weights calculated using the above procedure are robust to minor perturbations of tree topology, especially those concerning deep branching events and short internal branches. As long as rearrangements and uncertainty involve only relatively short paths on the tree, the weights are not affected substantially. The total number of the available archaeal genomes (168) was used to define the weight of the root. On this scale, the weights of individual genomes (Supplementary Table S1) varied from 0.016 (*Methanosarcina mazei* Go1) to 3.9 (*Candidatus* Korarchaeum cryptofilum OPF8).

### 2.3. Clade Representation in arCOGs

For the coarse-grained analysis of the distribution of various features across archaea, we defined 13 clades that roughly correspond to “classes” within archaeal phyla (Supplementary Table S1). Phyla with a low number of available genome sequences (Korarchaeota, Nanoarchaeota and Thaumarchaeota) were not subdivided; Candidatus “*Caldiarchaeum* *subterraneum*” genome, which represents the candidate phylum Aigarchaeota, was merged into Thaumarchaeota; *Methanopyrus kandleri* AV19, the only known representative of Methanopyri, was merged into Methanobacteria. Clade names used in this work are purely operational, do not represent formally accepted taxonomy, and do not necessarily follow conventional rules of nomenclature.

Representation of clades in arCOGs was calculated by normalizing the sum of the weights of genomes that encompass genes assigned to this particular arCOG by the sum of the weights of all genomes in the given clade.

The arCOGs that display a pattern of (near) exclusive presence in a particular set of clades were identified using the following index: *R*_E_ = (min *C_i_* + 0.0001)/(max *C_j_* + 0.0001) where *C_i_* is the number of members of the *i*-th clade in a given arCOG; the minimum is taken across all clades that belong to the analyzed set and the maximum is taken across all clades that do not belong to the set.

### 2.4. Reconstruction of Archaeal Phylogeny Using arCOGs

One thousand four hundred and two arCOGs with at least four clades represented by more than half of the genomes (representation calculated using genome weights as described above) were selected for phylogenetic analysis. Multiple alignments were constructed from the protein sequences of each arCOG; poorly aligned and fragmented sequences were removed from the alignments and clade representation was re-calculated for the sequences remaining in the alignments. Sequences from clades with less than 0.5 representation were also removed from the alignment. This procedure yielded 1366 arCOGs with at least four well-represented clades. Approximate ML phylogenetic trees were reconstructed from these alignments and rooted using the modified midpoint procedure [51].

Phylogenetic affinities of archaeal clades were assessed in these trees using a customized, automated procedure. For each clade present in these trees, all tree nodes that satisfy the following criteria were identified:

- weights of leaves descending from a node include at least 0.75 of the total weight of the given clade;
- among the leaves descending from a node, at least one other clade is represented by at least 0.75 of the total weight of this clade;
- among the internal nodes descending from a node, there is no other node satisfying the two criteria described above.

Under this procedure, the node selected to represent the phylogenetic affinity of the given clade is the shallowest node that covers most of the genomes from that clade and includes at least one of the clades that are its closest relative in this tree. The existence of such a node is guaranteed by the fact that the leaves descending from the root represent 100% of the clades in the given tree, of which there are at least four. If a clade is represented in the given arCOG by deep paralogs, there could be more than one node satisfying the above criteria; in these cases, the results are averaged across all such nodes.

The following formalism is used to calculate the phylogenetic affinity of a clade at the selected node of the tree. Any internal node of the tree ***N*** (including the root node ***R***) defines a set of descendant leaves {***L**_N_*}. An *i*-th leaf (*i*∈{***L**_N_*}) belongs to a clade *c_i_* and has a weight of *w_i_^ci^*. For the target clade c* and the selected node N* one can calculate the representation of sister clades as W_i_* = Σ*w_j_^i^*/Σ*w_k_^i^*, where *j*∈{**L**_N*_}, *k*∈{***L_R_***} and *i* ≠ c*. Normalization of W_i_* across the entire set of sister clades (R_i_* = W_i_*/ΣW_k_*, *k* ≠ c*) shows the phylogenetic affinities of the given clade in terms of other clades. Numbers close to 1 indicate that most neighbors of the given clade belong to a particular clade, suggesting sister relationships; series of smaller, almost equal, numbers suggest that the given clade is nearly basal to the subtree that includes these clades.

## 3. Results and Discussion

### 3.1. Update of the arCOGs

The updated arCOG database includes proteins from 168 complete archaeal genomes [52]. Altogether, 369,725 protein-coding genes (94% of the total gene complement) were assigned to 13,443 clusters. Furthermore, 99 protein sequences were added to the database by translating the genomic sequences, 50 of these (mostly ribosomal proteins) during this revision. High coverage of individual genomes had already been attained in the 2012 version of the arCOGs (91% of the protein-coding genes, on average) [11] and increased to 94% in the present update. The increase in most cases was due to the appearance of closely related genomes for several species. The biggest gainers are *Vulcanisaeta* species (+9%); *Aeropyrum* (+9%), *Methanocella* (+8%), *Nitrosoarchaeum* and *Nitrosopumilus* (about +7% on average) (see Supplementary Table S1). Although two Nanoarchaeal genomes are currently available and notwithstanding the tiny size of these genomes, their coverage remains the lowest at ~74%. Anomalous low coverage was also observed for the genome of *Methanosarcina mazei* Tuc01 (coverage 84%), which is closely related to *Methanosarcina mazei* Go1 (coverage 96%) but encompasses numerous small ORFs, most of which are products of multiple frameshifts of longer proteins present in *Methanosarcina mazei* Go1 and could result from pseudogenization and/or sequencing errors.

Analysis of gene frequencies (or more precisely, the representation of orthologous gene clusters) in prokaryotes reveals a universal pattern. At all evolutionary depths, the distribution of gene frequencies can be decomposed into three distinct components: the small core of (nearly) universal genes, the much larger “shell” of moderately conserved genes, and the large, incompletely defined “cloud” of rare genes [53]. In the previous update of the arCOGs, we concluded that the core and shell gene sets have already mostly saturated [11]. Thus, not surprisingly, the new estimates are very close to those obtained in the previous work, ~207 and ~2200 families, respectively (Supplementary Figure S1). Predictably, the cloud is still growing, and is now estimated to contain ~8900 families, up from ~7400 in 2012.

**Figure 1:**
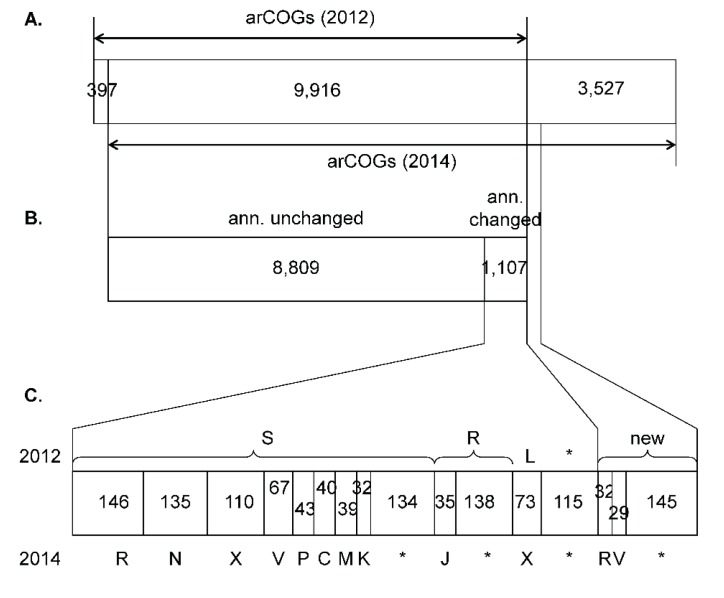
Changes in arCOGs between 2012 and 2014. (**A**) arCOGs added to and removed from the 2012 set; (**B**) Annotation changes and additions; (**C**) Breakdown of annotation changes into arCOG functional categories. 2012 annotations are indicated on top, 2014 annotations on the bottom. Functional categories are as follows. Information storage and processing: **J**, Translation, ribosomal structure, and biogenesis; **K**, Transcription; **L**, Replication, recombination, and repair. Cellular processes and signaling: **V**, Defense mechanisms; **M**, Cell wall/membrane/envelope biogenesis; **N**, Cell motility, secretion, and vesicular transport; **X**, Mobilome. Metabolism: **C**, Energy production and conversion; **P**, Inorganic ion transport and metabolism. Poorly characterized: **R**, General function prediction only; **S**, Function unknown. Asterisks indicate other categories combined.

In the new version of the arCOGs, many clusters that encompassed only a few genomes and consisted of genes that had paralogs in other, larger arCOGs were merged into the latter, resulting in elimination of 397 old arCOGs (Figure 1). Addition of 48 new genomes resulted in the generation of 3527 new arCOGs, several of which were manually rearranged on the basis of phylogenetic analysis. Notably, these changed arCOGs included the archaeal DNA replicative polymerases of the PolB family that were now manually divided into five arCOGs according to recent comparative genomics and phylogenetic analysis [22]: arCOG15271, PolB1; arCOG00329, PolB2; arCOG00328, PolB3; arCOG04926, DNA polymerase elongation subunit, PolB family; and arCOG15272, Casposon-associated protein-primed PolB family polymerase. Similarly, another family of essential proteins involved in DNA replication, the GINS, were split into separate clusters GINS15 (arCOG00551) and GINS23 (arCOG00552). Thus, the updated arCOG database reflects the evolutionary relationships and functional diversification of these key proteins as accurately as is currently feasible.

In addition to the previously defined 20 functional categories, the updated arCOGs include a new functional group “Mobilome” [38]. The mobilome includes proteins derived from viruses, plasmids, and transposable elements. Many of the arCOGs that comprise the mobilome were transferred from the “Replication, recombination, and repair” category but most were identified in the course of our recent work in which we analyzed (putative) viruses and other mobile elements integrated into archaeal genomes [19] (Figure 1). Another major change is the annotation of many previously uncharacterized arCOGs associated with diverse secretion systems in archaea (the respective arCOGs were moved to category N, “Motility, secretion, and vesicular transport”). These functional predictions were obtained in the course of a comprehensive comparative genomic analysis of Type IV-like secretion system genes and other conserved membrane proteins in archaea ([54] and KSM, YIW, and EVK, manuscript in preparation). Furthermore, for many arCOGs that remained in the same functional category, the annotation was refined. For example, numerous diverged components of the CRISPR-Cas system [15,16,55,56] are now provided with specific annotations in the arCOGs, and the same applies to several other defense systems that have recently been investigated in detail [20,56].

Several other new features were added to the release of the arCOG database file set [52]. These include the links from arCOGs to confidently matching pfam, CDD, and tigrfam IDs, prediction of transmembrane segments and signal peptides for each protein, and more (see README file in [52] for details). Furthermore, the arCOGs now include a higher hierarchical level of classification. In cases of significant sequence similarity between two or more arCOGs, they are combined into a supercluster. These superclusters are designed to be on the COG level (less than a family) and often include paralogous families in archaea that would be assigned to one COG if combined with bacteria. In these cases, the superclusters are given the respective COG name. Currently, the largest supercluster is COG0500, S-adenosyl-methionine-dependent methyltransferases (63 arCOGs), with only a few methylases with established substrate specificity. The largest superclusters among proteins without counterparts in the COGs are SC.00069, HEPN domain-containing proteins (5 arCOGs), SC.00027, Uncharacterized membrane protein DUF1673 family expanded in Methanosarcina species, and SC.00178, Uncharacterized protein, expanded in Halobacteria (4 arCOGs each). In addition, some superclusters include arCOGs with low sequence similarity that nevertheless have been shown to be highly diverged orthologs by additional analysis. Such examples include derived orthologs of the essential DNA replication protein CDC45 in crenarachaea [14], the OxaA/SpoJ/YidC translocases/secretases [54], and others.

Considering that genome annotation is one of the main applications of the arCOGs, we have assessed the state of annotation of genes that belong to specifically annotated arCOGs (*i.e.*, arCOGs in all categories other than “S, Uncharacterized proteins”) in the respective genome submissions in the RefSeq database. We found that ~22% of proteins in these arCOGs lack meaningful annotation in the databases (*i.e.*, are listed as “hypothetical” or “uncharacterized”). Furthermore, there are many examples when more than 90% of the proteins that belong to a functionally annotated arCOG are marked as “hypothetical” in public protein databases. These poorly annotated proteins included essential components of the translation apparatus such as large ribosomal subunit proteins L45 and L47 [57], pre-rRNA processing, and ribosome biogenesis “NOL1/NOP2/fmu family” (pfam13636 and [58]). Similarly, certain essential components of the replication system often remain annotated such as members of arCOGs that include GINS proteins and RecJ/CDC45 superfamily proteins in crenarchaea, which are the components of the CMG complex [13,14,59]. The same poor state of annotation was noticed for most of the components of the ESCRT system involved in archaeal cell division and membrane remodeling [21]. The common theme among these poorly annotated proteins is the weak sequence similarity between orthologs (often associated with the small size of the respective proteins), which means the annotations are not transferred by the commonly used genome annotation pipelines. The annotation of such diverged proteins would substantially benefit from consistent use of the arCOGs, and in particular arCOG-associated PSSMs, for genome annotation.

### 3.2. Analysis of the Shared Features of the Thermococci with Methanococci, Methanobacteria, and Methonopyrus

There is a continuing and still intense debate on whether or not a tree of life (species tree) is an adequate representation of the evolution of archaea and bacteria ([60,61,62,63,64,65,66,67,68] and references therein). The underlying cause of these persistent disagreements is the difference between the topologies of the phylogenetic trees for different genes. Nevertheless, although there are very few phylogenetic trees with identical topologies, the phylogenies of the conserved and particularly (nearly) universal gene trees in archaea and bacteria are substantially congruent, suggesting that a consensus topology of such trees could represent a central trend in prokaryotic evolution. The existence of such a central trend justifies attempts to construct a “statistical tree of life” by phylogenetic analysis of subsets of the nearly universal genes that have been selected using varying criteria and different phylogenetic methods [10,12,50,68,69,70,71,72].

The putative global species trees of archaea and bacteria show several points of instability for which it is hard to determine a reliable branching order, with different approaches often yielding conflicting results. When it comes to the archaeal phylogeny, such persistent uncertainties include the position of the Nanoarchaea [24,50,73,74,75], the position of *Methanopyrus kandleri*, the monophyly of class I methanogens [36,71,72,76,77,78], and the validity of the TACK superphylum (an assemblage of archaeal phyla that includes Taumarchaeota, Crenarchaeota, Korarchaeota, and *Candidatus* Caldiarchaeum subterreneum, only known the representative of the potential phylum Aigarchaeota) [12,50,79,80]. Furthermore, the phylogenetic affinities of several derived archaea with small genomes, such as ARMANs and Nanosalina, remain uncertain [71,81,82].

Despite being the primary approach for the study of gene evolution, phylogenetic analysis is fraught with several notorious methodological shortcomings, such as sensitivity to the number and quality of the alignment positions, species sampling bias, and long or short branch attraction [83,84,85]. Different methods of tree reconstruction often produce incompatible topologies, and alternative tree topologies are often statistically indistinguishable. A complement to the traditional, sequence-based molecular phylogeny is the search for shared derived characters (synapomorphies) that has the potential to circumvent the above caveats. Here we apply a combination of phylogenetic analysis and the search for shared genes by using the phyletic patterns (patterns of gene presence/absence in archaeal lineages) of the arCOGs to shed light on the phylogenetic affinity of the Thermococci.

Until recently, the basal position of Thermococci in the euryarchaeal part of the archaeal tree remained undisputed. This position of the Thermococcal branch had been reproduced with a variety of phylogenetic methods, gene sets, and species samples [12,24,50,72,86]. However, all these analyses overlapped in that each of them included a significant fraction of genes encoding components of the translational apparatus. In a recently reported phylogeny of archaeal genes involved in DNA replication, the Thermococci were confidently grouped with class I methanogens [71]. The authors suspected that this clustering could be a tree reconstruction attraction artifact but several alternative reconstructions (e.g., different combinations of taxa) and the use of different amino acid similarity matrices have consistently produced the same clustering of these lineages [71]. A separate phylogenetic tree of family B DNA polymerases only (specifically, the PolB3 branch) also confidently groups these lineages [22]. Furthermore, Thermococci and Methanococci share two intein insertions in the same positions in this DNA polymerase gene, whereas Methanobacteria possess two split genes that most likely emerged via trans-splicing of ancestral inteins [22]. However, inteins are also present in several halobacterial PolB3 which have three intein insertions, two in positions shared with Thermococci and Methanococci—although the halobacterial inteins substantially differ from those in the other groups in sequence and length. Taken together, these observations are better compatible with the monophyly of Thermococci, Methanococci, and Methanobacteria, although alternative scenarios, such as HGT of the PolB3 genes between these archaeal groups, or differential loss of inteins in other groups of archaea, cannot be ruled out.

**Figure 2:**
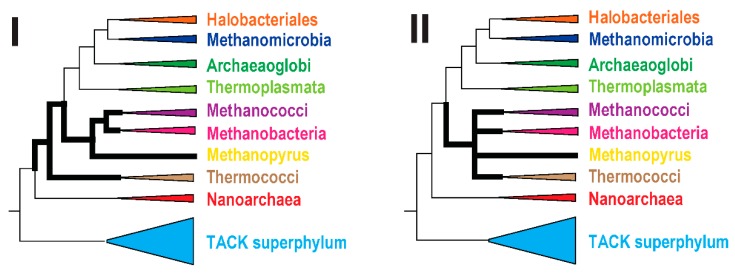
Alternative phylogenies for the Themococci Thick lines show the relationships between Thermococci and their closest relatives, Methanococci, Methanobacteria, and Methanopyrus. Alternative topologies of Thermococci in the Euryarchaeota tree are indicated with Roman numerals (I and II). The topology II is shown as multifurcation because the specific order of branching of these lineages has not been addressed or analyzed in this work.

Prompted by these observations, we sought evidence in support of either the basal position of the Thermococci in the Euryarchaea tree (topology I) or their clustering with class I methanogens (topology II) (Figure 2). First, we reconstructed 1366 phylogenetic trees for arCOGs that have at least four well-represented clades (see Methods for details; the trees are available on [87]); 951 of these trees contained the Thermococci clade. For each of these 951 trees, the affinity of the Thermococci to other clades was quantified (see Methods for details). Ranked by the average affinity, the most common neighbors of the Thermococci were Methanococci (0.49), followed by Korarchaeota (0.48) and Nanoarchaeota (0.44) (see Supplementary Table S2).

**Figure 3:**
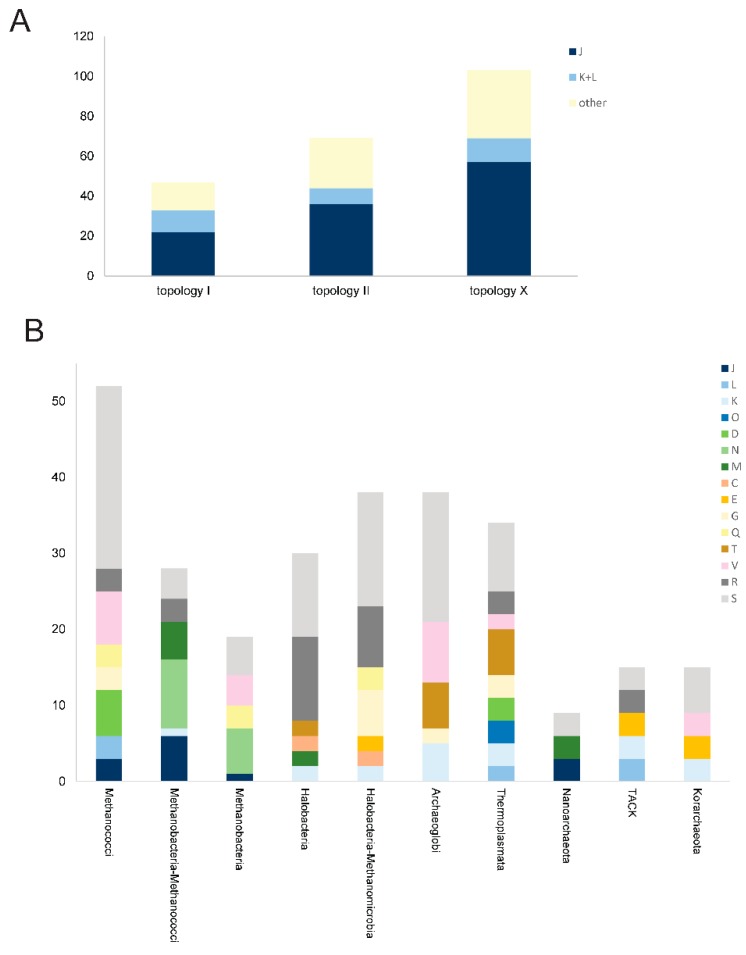
Phylogenomic analysis of the Thermococci. (**A**) Breakdown of the phylogenies for the 219 core arCOGs. Letters indicate the functional categories for the respective arCOGs; (**B**) Breakdown of the arCOGs that are exclusively shared between Thermococci and other archaeal lineages. The vertical axis indicates the contribution of shared arCOGs. Individual arCOGs contribute 1 to 3 units depending on their exclusivity ratio (see Methods and Supplementary Table S3). Letters indicate functional categories of arCOGs. Thermococci were compared with individual major lineages and, in two cases, with pairs of lineages related according to phylogenetic analyses.

Among the 219 arCOGs that belong to the archaeal core (defined here as arCOGs with all clades, excluding Nanoarchaeota, represented by at least 0.75 genomes, under the weighting scheme described in the Methods), 47 trees support topology I (basal position of Thermococci), 69 support topology II (a clade of Thermococci with Methanococci and/or Methanobacteria) (Figure 3A), and the remaining 103 trees are not fully compatible with either (e.g., Thermococci associated with non-euryarchaeal lineages). Among the trees that support topology II, 36 belong to the translation system, indicating that phylogenetic analysis of this system yields a mixed signal. Some earlier studies (e.g., [78,88]) produced trees, supporting topology II using a limited taxonomic sample that was available at the time. Nowadays this topology is often considered to be a long branch attraction artifact given that topology I appears more consistently. There is no single clearly dominant topology in the “topology X” category (Figure 3A).

We then analyzed the genes that are (almost) exclusively shared by Thermococci and each of the other major archaeal lineages and higher order groups, such as Halobacteria-Methanomicrobia, Methanobacteria-Methanococci, the TACK superphylum, and others (Supplementary Table S3). We selected only the arCOGs with the exclusivity ratio *E*_R_ > 10 (see Methods). There are only few such arCOGs for each pair of the considered lineages, and most of them belong to functional categories R and S (poorly characterized genes). The largest number of such genes are shared by Thermococci and Methanococci (Figure 3B). Among the better characterized arCOGs, Thermococci exclusively share with Methanococci slightly more genes from the informational functional categories (J, L, K, and O) and division-, secretion-, and membrane biogenesis-related genes (N, D, M) than with other lineages. This result could be explained by a single event of gene loss in the branch that includes all euryarchaea, to the exclusion of the Thermococci (topology I), rather than by the emergence of these genes in the Thermococci/class I methanogens branch (topology II). Nevertheless, we examined in greater detail the features of these genes, especially those that have the highest exclusivity ratio.

**Figure 4:**
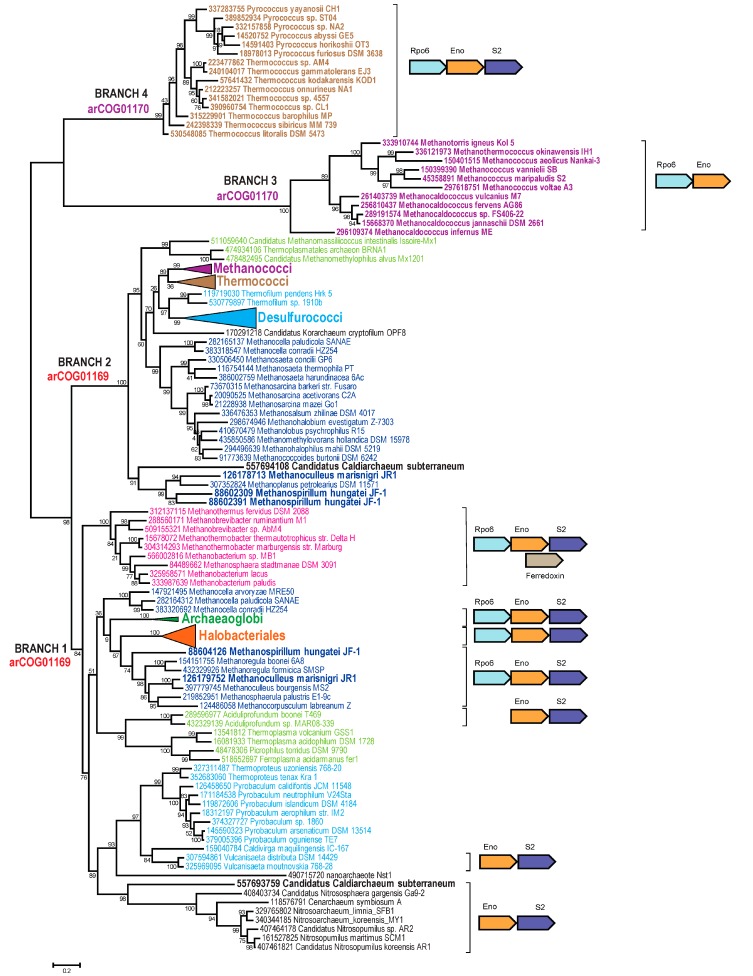
Phylogenetic analysis of the archaeal enolase family. The MUSCLE program [46] was used for construction of sequence alignment. The approximate maximum likelihood tree was reconstructed using the FastTree program [49] (178 sequences and 410 aligned positions). The sequences are denoted by their GI numbers and species names. Several branches are collapsed and shown as triangles denoted by the respective lineage taxonomy name. The complete tree is available in Supplementary file S4. Color code is the same as in Figure 2. Species or lineages that have paralogs elsewhere in the tree are in bold. The conserved neighborhoods (if any) are shown on the right side of the tree for the respective branches. Homologous genes are shown by arrows of the same color. The arCOG numbers are provided for major branches. Abbreviations: Rpo6: DNA-directed RNA polymerase subunit K/omega and S2: RpsB, ribosomal protein S2.

A well characterized protein in this group is enolase, a key glycolytic enzyme that additionally has been identified as a subunit of the bacterial RNA degradosome [89,90]. The enolase is often encoded in a conserved gene neighborhood within the ribosomal superoperon [91] and in the majority of the archaeal and bacterial genomes is present in a single copy. In arCOGs, enolase is represented by arCOG01169 and arCOG01170, which together belong to the supercluster COG0148. All Methanococci and Thermococci, several Metanomicrobia, and *Candidatus* Caldiarchaeum subterraneum have two enolase paralogs. We reconstructed the phylogenetic tree for the enolases of both arCOGs and overlaid the genomic contexts of the respective genes (Figure 4).

Phylogenetic analysis reveals four major branches in the enolase tree (Figure 4, branches 1–4). Three of these (branches 1, 3, and 4) share a mostly conserved context within the ribosomal superoperon. Specifically, these enolase genes are located next to genes coding for DNA-directed RNA polymerase subunit Rpo6 and ribosomal protein S2, each of which is present in a single copy per genome in most archaea. In contrast, branch 2 genes are scattered across the genome without any contextual conservation outside of the narrow clades. However, branch 2 proteins clearly cluster in the tree with branch 1, to the exclusion of branches 3 and 4. Branch 2 shows Methanococci and Thermococci as sister groups, although overall the topology in branches 1 and 2 is poorly compatible with the core phylogeny of Archaea.

Several evolutionary scenarios as well as persistent tree reconstruction artifacts can be invoked to explain the observed relationships among archaeal enolases. Evolution of enolases in archaea clearly involved at least two ancient duplications or acquisitions via HGT that produced (pseudo)paralogs in some groups and several likely xenologous gene displacements that shuffled the clades in branches 1 and 2. The disagreement between the gene context similarity and sequence conservation also make genomic rearrangements a distinct possibility.

One group of evolutionary scenarios is based on the premise that branches 1 and 2 represent the ancestral enolase, whereas branches 3 and 4 are (pseudo)paralogs present in Methanococci and Thermococci. If the tree topology is taken at face value, one would have to postulate an acquisition of a distinct enolase gene from a source outside of the available archaeal tree. Because genes in branches 3 and 4 share the context with branch 1 genes, this scenario would require subsequent intragenomic rearrangement, which would place the acquired genes into the (supposedly preferred) location within the ribosomal superoperon, displacing the original gene copies to a different location. Clearly, this is not a parsimonious scenario.

Another group of scenarios posits that the (pseudo)paralogs evolved by duplication within the respective ancestral genome or by acquisition from a closely related organism so that the basal position of branches 3 and 4 is a tree reconstruction artifact caused by acceleration of evolution. Under this scenario, the paralog retaining the ancestral context underwent a period of fast evolution probably associated with a functional shift whereas the second, slowly evolving paralog most likely retained the original function while losing the original context. In more general terms, this parsimonious scenario of enolase evolution corresponds to the subfunctionalization route of evolution whereby after gene duplication, the two paralogs retain complementary subsets of the ancestral gene functions while undergoing evolutionary acceleration caused by partial loss of constraints [92]. In the case of enolase, it appears most likely that proteins in branch 2 retained the glycolytic function but lost the putative ancillary function linked to ribosomal biogenesis and dependent on the ancestral genomic context. Conversely, the enzymes in branches 3 and 4 retain only the ancillary function but not the role in glycolysis.

With respect to the evolutionary relationships between Methanococci, Methanobacteria, and Thermococci, these scenarios prompt the question how the similarity of the inferred histories of Methanococci and Thermococci, to the exclusion of Methanobacteria, came to be. If Thermococci are basal to all Euryarchaeaota (topology I), these events had to have happened in the respective ancestors of Methanococci and Thermococci independently. Alternatively, under topology II, the duplication, acquisition, or rearrangement event(s) either occurred in the common ancestor of Methanococci, Methanobacteria, and Thermococci with Methanobacteria subsequently losing the novel features; or Methanococci and Thermococci share a more recent common ancestor compared to Methanobacteria. The latter route of evolution appears especially likely under the subfunctionalization scenario outlined above, which involves dramatic acceleration of evolution in the *in situ* paralog of enolase because in this case no loss of this gene in Methanobacteria is required.

**Figure 5:**
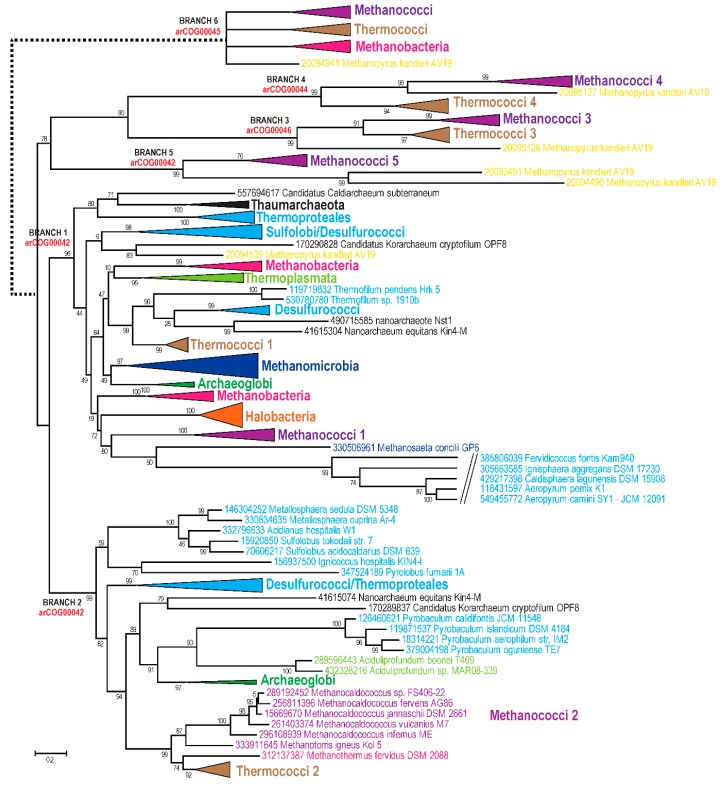
Phylogenetic analysis of archaeal TilS/MesJ family. The tree was reconstructed as described in Figure 4; 284 sequences and 285 aligned positions were used. The complete tree is available in Supplementary file S4. Coloring scheme is the same as in Figure 2. Sequences and collapsed branches are shown as in Figure 4.

The COG00037 supercluster consists of three arCOGs (arCOG00042, arCOG00044, and arCOG00046). All these proteins belong to the TilS/MesJ family of tRNA(Ile)-lysidine synthases, members of the *N*-type ATP pyrophosphatase superfamily, found in all three domains of life [93], and are involved in an essential modification of tRNA(Ile) [94]. Two of these arCOGs are exclusively shared by Thermococci with class I methanogens. The phylogenetic tree for this supercluster (Figure 5) revealed an even more complicated picture than the enolase tree. Apparently, evolution of this family was affected by a number of evolutionary events including HGT, duplications, and accelerations of evolutionary rate. However, some interpretations appear straightforward. Most arCOG00042 representatives belong to two major branches (branches 1 and 2) that probably evolved via an ancestral duplication, although a scenario with multiple HGT events cannot be ruled out (Figure 5). Methanococci are present in both of these branches but also form three additional branches, often together with representatives of other lineages of class I methanogens that apparently encompass fast-evolving variants of this gene. Branch 3 and branch 4 correspond to arCOG00046 and arCOG00044, respectively. The clustering of Thermococci with Methanococci is observed in three branches (2, 3, and 4) (Figure 5). Under topology I, these observations imply that these three genes were acquired by Thermococci from class I methanogens via three independent HGT events. Alternative hypotheses compatible with topology I would involve a long branch attraction artifact for several paralogs that would have to be assumed to experience acceleration of evolution independently in both Thermococci and class I methanogens or multiple gene losses of 4 (or more) ancestral paralogs in all other branches of archaea. Scenarios based on topology II imply that the duplications occurred in the common ancestor of Thermococci and class I methanogens and gave rise to two or three fast-evolving TilS/MesJ family paralogs. An apparent distant homolog of the TilS/MesJ family, arCOG00045, is also shared between Thermococci and class I methanogens, to the exclusion of other archaea. This arCOG belongs to a different COG (COG01365), and, in addition to Thermococci and class I methanogens, is found only in a few bacterial genomes [38]. The functions of these proteins are not known but most of them are fused to a KH RNA-binding domain [95], implicating these enzymes in RNA modification. This family is likely to represent yet another divergent paralog resulting from duplications in the common ancestor of the same clade (Figure 5).

Many genes (Figure 3B, Supplementary Table S3) are specifically shared by the Thermococci and class I methanogens that belong to functional categories D (Cell cycle control, cell division, chromosome partitioning) and N (Motility, secretion, and vesicular transport). This group includes several genes that are associated with the ESCRT-III system, predicted to be involved in cell division and/or membrane remodeling [21]. The details on the organization of the respective loci and the presence of highly similar gene neighborhoods in Thermococci and Methanococci have been discussed previously [21]. Furthermore, Thermococci and class I methanogens share a distinct type IV-like secretion system that is centered around specific pili assembly ATPase (arCOG01819) [96] and prepilin signal peptidase EppA (arCOG02300) [97]. Notably, the ATPases of arCOG01819 in both class I methanogens and Thermococci share an intein insertion in the same position.

Several genes that are shared by Thermococci and class I methanogens do not pass the exclusivity threshold, being present in some other archaea (often Korarchaeum), but share similar features and group together in the respective phylogenetic trees. Two such genes are involved in DNA replication, namely RPA14, a subunit of the ssDNA-binding protein RPA complex (arCOG05741) and Orc1/Cdc6 homolog, an AAA superfamily ATPase (arCOG00472) [18,71]. Both of these genes show increased evolutionary rates in both Thermococci and class I methanogens.

Identification of arCOGs with phyletic patterns supporting topology I is more problematic because this topology includes Thermococci as a basal branch. Nevertheless, we searched for genes that are highly conserved in Thermococci and are shared with all other euryarchaeal lineages except for class I methanogens; this pattern implies a single gene loss in the class I methanogen branch and thus is best compatible with topology I. No genes with this pattern that would pass the exclusivity threshold were identified. Alternatively, we searched for Thermococci genes shared with the TACK superphylum but missing in other euryarchaea; this pattern implies a single loss for topology I and at least two losses for topology II depending on the branching pattern of the four major lineages within the euryarchaeal clade. We found five genes with this pattern (Figure 3B, Supplementary Table S3). Only one of these genes encodes an essential informational protein, the DNA replication initiation complex subunit of the GINS23 family (arCOG00552) [13,59].

## 4. Conclusions

With the accelerating accumulation of genome sequences from diverse groups of archaea and bacteria, nearly all genes in most organisms can be included in clusters of orthologs, which makes collections of such clusters increasingly efficient tools for genome annotation and phylogenomic analysis. Clearly, an essential requirement of such tools is accuracy of orthology identification and careful functional annotation of the clusters. With the arCOGs, a consistent attempt to achieve these goals was made through a combination of amended procedures for automatic clustering and intensive manual curation, aided by incorporation of the results of multiple research projects. The new arCOGs also include hierarchy in the form of superclusters that more realistically capture the evolutionary relationships between genes compared to disconnected arCOGs. Further developments are expected to include continued refinement of arCOGs, in particular by systematically applying phylogenetic analysis to resolve complex relationships within gene families with multiple paralogs. Assessment of the current archaeal genome annotation in public databases indicates that consistent use of arCOGs can significantly improve the annotation quality.

We are particularly interested in the phylogenomic applications of arCOGs aimed at comprehensive reconstruction of the evolutionary relationships between different groups of archaea and other organisms. Clearly, these relationships cannot be adequately captured by a phylogenetic tree of a single universal gene (e.g., 16S rRNA) or even a small group of topologically consistent phylogenetic trees such as those of multiple translation system components. Even if such trees show non-random topological similarity to the trees of many other genes [98], they remain “trees of 1%” [67]. In order to actually reconstruct genome and organismal evolution, phylogenomic analysis is essential. Here we employed the arCOGs in their capacity as a phylogenomic platform to reassess the evolutionary status of the Thermococci, which are traditionally viewed as the basal branch of the Euryarchaeota. Two features of arCOGs (and other collections of clusters of orthologs) underlie their utility for phylogenomics: (i) accurate identification of orthologous gene sets, which is a prerequisite for informative phylogenetic analysis; and (ii) straightforward extraction of phyletic patterns of any gene, which provides for identification of putative derived shared characters (synapomorphies). We applied both these approaches to the phylogenomic study of the Thermococci and in both cases detected a signal of evolutionary affinity with the Methanococci and Methanobacteria although a weaker signal corresponding to a basal position of the Thermococci in the Euryarchaea tree was detected as well. Clearly, all genomes have mixed legacy so that their evolution cannot be represented by a single position in a tree (even if it is a consensus of numerous trees), as emphasized, for instance, by recent observations indicating that the emergence of several major groups of archaea could have been triggered by an influx of bacterial genes [69]. Nevertheless, the findings reported here strongly suggest that the Thermococci share substantial common history with class I methanogens, which could involve shared ancestry, with vertical descent, extensive HGT, or both. We expect arCOGs to be instrumental for phylogenomic dissection of the evolution of other groups of archaea as well.

## Supplementary Files

Supplementary File 1
