# Supplementary material for: Archaeal Clusters of Orthologous Genes (arCOGs): An Update and Application for Analysis of Shared Features between Thermococcales, Methanococcales, and Methanobacteriales
Source: Life (Basel). 2015 Mar 10;5(1):818–40. doi: 10.3390/life5010818 (PMC4390880; doi:10.3390/life5010818)
Supplement: Supplementary File 1 [file life-05-00818-s001.zip › Supplementary Figure S1.pdf]

Supplementary

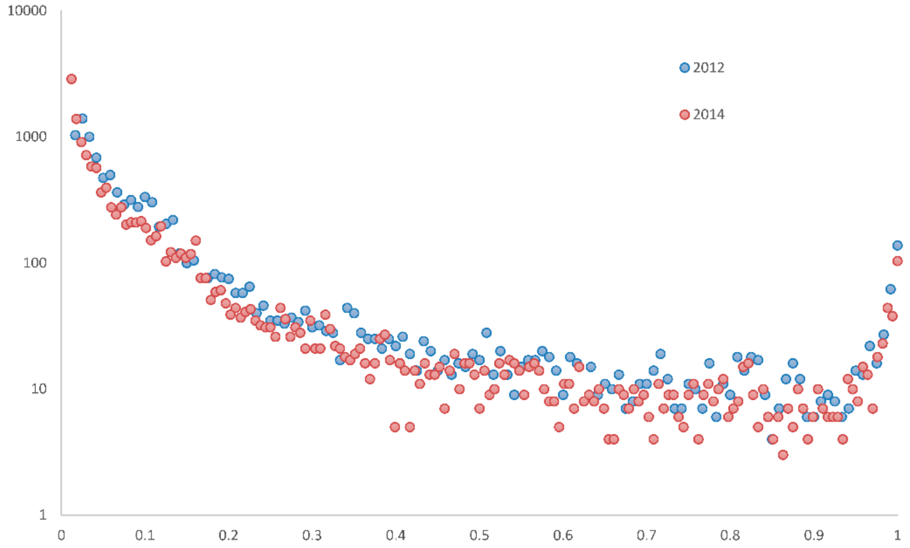

Figure S1. Commonality plot comparison for arCOGs2012 and 2014 versions of database. Circles show the number of arCOGs(vertical axis) that include the given number of distinct genomes (horizontal axis). Both axes are in the log scale.
