# Supplementary material for: Archaeal Clusters of Orthologous Genes (arCOGs): An Update and Application for Analysis of Shared Features between Thermococcales, Methanococcales, and Methanobacteriales
Source: Life (Basel). 2015 Mar 10;5(1):818–40. doi: 10.3390/life5010818 (PMC4390880; doi:10.3390/life5010818)
Supplement: Supplementary File 1 [file life-05-00818-s001.zip › Supplementary File S4.pdf]

Supplementary File S4. Phylogenetic trees in Newick format

Enolase family

((530548085|Therm|Thermococcus_litoralis_DSM_5473:0.03351000,(242398339|Therm|Thermococcu
s_sibiricus_MM_739:0.10515000,(315229901|Therm|Thermococcus_barophilus_MP:0.08318000,((337
283755|Therm|Pyrococcus_yayanosii_CH1:0.07184000,(389852934|Therm|Pyrococcus_sp-
_ST04:0.06082000,((332157858|Therm|Pyrococcus_sp-
_NA2:0.07831000,(14520752|Therm|Pyrococcus_abyssi_GE5:0.07273000,14591403|Therm|Pyrococcu
s_horikoshii_OT3:0.11154000)0.59:0.01476000)0.18:0.03442000,18978013|Therm|Pyrococcus_furiosu
s_DSM_3638:0.14454000)0.09:0.02572000)1.00:0.13225000)1.00:0.08744000,((223477862|Therm|The
rmococcus_sp-
_AM4:0.01370000,240104017|Therm|Thermococcus_gammatolerans_EJ3:0.01965000)1.00:0.0961200
0,(57641432|Therm|Thermococcus_kodakarensis_KOD1:0.15385000,(212223257|Therm|Thermococcu
s_onnurineus_NA1:0.02130000,(341582021|Therm|Thermococcus_sp-
_4557:0.02403000,390960754|Therm|Thermococcus_sp-
_CL1:0.03827000)0.76:0.02588000)0.61:0.01931000)0.96:0.04343000)0.90:0.04544000)0.96:0.0623100
0)0.97:0.05175000)0.43:0.04473000)0.99:0.74535000,(296109374|Metha|Methanocaldococcus_infern
us_ME:0.32498000,((333910744|Metha|Methanotorris_igneus_Kol_5:0.13640000,((336121973|Metha
|Methanothermococcus_okinawensis_IH1:0.12637000,150401515|Metha|Methanococcus_aeolicus_N
ankai-
3:0.34179000)1.00:0.13106000,((150399390|Metha|Methanococcus_vannielii_SB:0.10458000,4535889
1|Metha|Methanococcus_maripaludis_S2:0.11853000)0.98:0.10191000,297618751|Metha|Methanoc
occus_voltae_A3:0.38050000)0.97:0.09311000)1.00:0.12495000)1.00:0.23841000,(261403739|Metha|
Methanocaldococcus_vulcanius_M7:0.13561000,(256810437|Metha|Methanocaldococcus_fervens_AG
86:0.04702000,(289191574|Metha|Methanocaldococcus_sp-_FS406-
22:0.03851000,15668370|Metha|Methanocaldococcus_jannaschii_DSM_2661:0.01640000)0.95:0.0397
5000)0.99:0.08668000)0.96:0.08006000)0.89:0.29217000)1.00:1.69311000,((((511059640|Metha|Cand
idatus_Methanomassiliicoccus_intestinalis_Issoire-
Mx1:0.28724000,(474934106|Therm|Thermoplasmatales_archaeon_BRNA1:0.08925000,478482495|u
ncla|Candidatus_Methanomethylophilus_alvus_Mx1201:0.09613000)1.00:0.30624000)1.00:0.21249000
,(((((297620182|Metha|Methanococcus_voltae_A3:0.16550000,(150400125|Metha|Methanococcus_v
annielii_SB:0.11725000,45357959|Metha|Methanococcus_maripaludis_S2:0.06304000)1.00:0.0796200
0)0.98:0.07878000,((296109288|Metha|Methanocaldococcus_infernus_ME:0.11366000,(261403736|M
etha|Methanocaldococcus_vulcanius_M7:0.03869000,(15668407|Metha|Methanocaldococcus_jannas
chii_DSM_2661:0.00731000,(289193285|Metha|Methanocaldococcus_sp-_FS406-
22:0.00947000,256810404|Metha|Methanocaldococcus_fervens_AG86:0.03610000)0.86:0.00695000)0
.94:0.01672000)0.89:0.02340000)1.00:0.09506000,(333910701|Metha|Methanotorris_igneus_Kol_5:0.
09464000,(150401040|Metha|Methanococcus_aeolicus_Nankai-
3:0.10759000,336122130|Metha|Methanothermococcus_okinawensis_IH1:0.08279000)0.99:0.060770
00)0.21:0.01983000)0.77:0.03541000)1.00:0.10097000,(((315231306|Therm|Thermococcus_barophilus
_MP:0.02679000,(242398778|Therm|Thermococcus_sibiricus_MM_739:0.02347000,530549604|Ther

m|Thermococcus_litoralis_DSM_5473:0.01994000)1.00:0.06014000)0.96:0.05472000,((212224764|The
rm|Thermococcus_onnurineus_NA1:0.03736000,((57642041|Therm|Thermococcus_kodakarensis_KOD
1:0.02826000,(223477394|Therm|Thermococcus_sp-
_AM4:0.05716000,240102135|Therm|Thermococcus_gammatolerans_EJ3:0.01084000)0.96:0.0215100
0)0.72:0.00502000,(341582557|Therm|Thermococcus_sp-
_4557:0.02083000,390961280|Therm|Thermococcus_sp-
CL1:0.00519000)0.93:0.01304000)0.85:0.01995000)0.67:0.01406000,(337284767|Therm|Pyrococcus
yayanosii_CH1:0.06485000,((18976587|Therm|Pyrococcus_furiosus_DSM_3638:0.03793000,38985181
4|Therm|Pyrococcus_sp-
_ST04:0.02168000)0.90:0.01206000,(14521916|Therm|Pyrococcus_abyssi_GE5:0.03183000,(14591684
|Therm|Pyrococcus_horikoshii_OT3:0.03029000,332158504|Therm|Pyrococcus_sp-
_NA2:0.01220000)0.92:0.01214000)0.94:0.02605000)0.97:0.03303000)0.37:0.03473000)0.90:0.034740
00)1.00:0.11245000,20095083|Metha|Methanopyrus_kandleri_AV19:0.35527000)0.36:0.05391000)0.8
9:0.06999000,((119719030|Therm|Thermofilum_pendens_Hrk_5:0.09358000,530779897|Therm|Ther
mofilum_sp-_1910b:0.13503000)1.00:0.14451000,(156937797|Therm|Ignicoccus_hospitalis_KIN4-
I:0.33641000,((389860362|Therm|Thermogladius_cellulolyticus_1633:0.20399000,((126466164|Therm
|Staphylothermus_marinus_F1:0.02513000,297527152|Therm|Staphylothermus_hellenicus_DSM_127
10:0.01781000)1.00:0.11530000,(296241919|Therm|Thermosphaera_aggregans_DSM_11486:0.186950
00,(320101232|Therm|Desulfurococcus_mucosus_DSM_2162:0.06990000,(218884751|Therm|Desulfu
rococcus_kamchatkensis_1221n:0.01168000,390937813|Therm|Desulfurococcus_fermentans_DSM_16
532:0.00827000)0.99:0.06053000)0.99:0.07221000)0.96:0.05797000)0.15:0.02927000)1.00:0.16995000
,((124027784|Therm|Hyperthermus_butylicus_DSM_5456:0.26901000,347524475|Therm|Pyrolobus_f
umarii_1A:0.24080000)0.74:0.05700000,(305663579|Therm|Ignisphaera_aggregans_DSM_17230:0.380
01000,((385805179|Therm|Fervidicoccus_fontis_Kam940:0.50329000,(((15897800|Therm|Sulfolobus_
solfataricus_P2:0.04812000,238619738|Therm|Sulfolobus_islandicus_M-16-
4:0.03064000)1.00:0.28171000,(332797012|Therm|Acidianus_hospitalis_W1:0.18539000,(15921475|T
herm|Sulfolobus_tokodaii_str-
_7:0.14367000,70607133|Therm|Sulfolobus_acidocaldarius_DSM_639:0.28325000)0.99:0.14091000)0.
90:0.07754000)0.59:0.05866000,(146304431|Therm|Metallosphaera_sedula_DSM_5348:0.13458000,3
30834423|Therm|Metallosphaera_cuprina_Ar-
4:0.18453000)0.99:0.14153000)1.00:0.27683000)0.99:0.19488000,((14602076|Therm|Aeropyrum_per
nix_K1:0.09362000,549456187|Therm|Aeropyrum_camini_SY1_-
_JCM_12091:0.11233000)1.00:0.32994000,(302348631|Therm|Acidilobus_saccharovorans_345-
15:0.16255000,429216495|Therm|Caldisphaera_lagunensis_DSM_15908:0.25935000)1.00:0.22903000)
0.70:0.08011000)0.94:0.09225000)0.97:0.10396000)0.97:0.08370000)0.36:0.03567000)1.00:0.0983400
0)0.98:0.07689000)0.26:0.03946000,170291218|Candi|Candidatus_Korarchaeum_cryptofilum_OPF8:0.
52092000)0.71:0.05544000,((282165137|Metha|Methanocella_paludicola_SANAE:0.08257000,383318
547|Metha|Methanocella_conradii_HZ254:0.05870000)1.00:0.26029000,((330506450|Metha|Methan
osaeta_concilii_GP6:0.12444000,(116754144|Metha|Methanosaeta_thermophila_PT:0.14997000,3860
02759|Metha|Methanosaeta_harundinacea_6Ac:0.15989000)0.42:0.04622000)1.00:0.12803000,((7367
0315|Metha|Methanosarcina_barkeri_str-
_Fusaro:0.01817000,(20090525|Metha|Methanosarcina_acetivorans_C2A:0.01158000,21228938|Meth
a|Methanosarcina_mazei_Go1:0.01379000)0.99:0.03740000)1.00:0.13338000,(336476353|Metha|Met
hanosalsum_zhilinae_DSM_4017:0.18031000,(298674946|Metha|Methanohalobium_evestigatum_Z-

7303:0.22140000,((410670479|Metha|Methanolobus_psychrophilus_R15:0.11216000,435850586|Met
ha|Methanomethylovorans_hollandica_DSM_15978:0.11229000)0.86:0.01415000,(294496639|Metha|
Methanohalophilus_mahii_DSM_5219:0.14204000,91773639|Metha|Methanococcoides_burtonii_DSM
_6242:0.10427000)0.83:0.02529000)0.63:0.01534000)0.05:0.01979000)0.95:0.05551000)1.00:0.117990
00)0.99:0.08540000)0.99:0.10511000)0.61:0.03484000)0.96:0.09848000,(557694108|uncla|Candidatus
_Caldiarchaeum_subterraneum:0.78086000,((126178713|Metha|Methanoculleus_marisnigri_JR1:0.292
88000,307352824|Metha|Methanoplanus_petrolearius_DSM_11571:0.20207000)0.95:0.10627000,(88
602309|Metha|Methanospirillum_hungatei_JF-
1:0.26180000,88602391|Metha|Methanospirillum_hungatei_JF-
1:0.21826000)0.83:0.07739000)1.00:0.20644000)0.91:0.07656000)1.00:0.42394000,((312137115|Meth
a|Methanothermus_fervidus_DSM_2088:0.27610000,((288560171|Metha|Methanobrevibacter_rumin
antium_M1:0.12424000,509155321|Metha|Methanobrevibacter_sp-
_AbM4:0.09828000)0.99:0.10191000,((15678072|Metha|Methanothermobacter_thermautotrophicus_
str-_Delta_H:0.02598000,304314293|Metha|Methanothermobacter_marburgensis_str-
_Marburg:0.01748000)1.00:0.14056000,(566002816|Metha|Methanobacterium_sp-
_MB1:0.10851000,(84489662|Metha|Methanosphaera_stadtmanae_DSM_3091:0.24116000,(3259585
71|Metha|Methanobacterium_lacus:0.05761000,333987639|Metha|Methanobacterium_paludis:0.073
18000)0.88:0.03460000)0.77:0.03388000)0.99:0.07517000)0.22:0.03381000)0.85:0.03823000)1.00:0.20
836000,((((147921495|Metha|Methanocella_arvoryzae_MRE50:0.14090000,(282164312|Metha|Meth
anocella_paludicola_SANAE:0.09311000,383320692|Metha|Methanocella_conradii_HZ254:0.08290000
)1.00:0.08995000)1.00:0.19460000,((288932312|Archa|Ferroglobus_placidus_DSM_10642:0.13320000,
(284161315|Archa|Archaeoglobus_profundus_DSM_5631:0.17000000,(488600173|Archa|Archaeoglob
us_sulfaticallidus_PM70-
1:0.18385000,(11498732|Archa|Archaeoglobus_fulgidus_DSM_4304:0.15287000,327401471|Archa|Ar
chaeoglobus_veneficus_SNP6:0.13295000)0.34:0.03548000)0.95:0.06250000)0.86:0.04914000)1.00:0.3
0494000,(((15790222|Halob|Halobacterium_sp-_NRC-
1:0.00000000,169235951|Halob|Halobacterium_salinarum_R1:0.00000000)0.00:0.21203000,(((((43363
8717|Halob|Halovivax_ruber_XH-70:0.07770000,((336252146|Halob|Halopiger_xanaduensis_SH-
6:0.02990000,(289580113|Halob|Natrialba_magadii_ATCC_43099:0.04063000,435847142|Halob|Natr
onococcus_occultus_SP4:0.06144000)0.87:0.02074000)0.85:0.01787000,(429192487|Halob|Natronoba
cterium_gregoryi_SP2:0.06896000,(284165816|Halob|Haloterrigena_turkmenica_DSM_5511:0.037630
00,(397772877|Halob|Natrinema_sp-_J7-
2:0.01532000,433590983|Halob|Natrinema_pellirubrum_DSM_15624:0.03034000)0.93:0.02073000)0.
04:0.01202000)0.80:0.01537000)0.99:0.05259000)0.99:0.04815000,((300711105|Halob|Halalkalicoccus
_jeotgali_B3:0.07865000,(257053579|Halob|Halorhabdus_utahensis_DSM_12940:0.02681000,5290786
94|Halob|Halorhabdus_tiamatea_SARL4B:0.00719000)1.00:0.15574000)0.96:0.04333000,(257388638|
Halob|Halomicrobium_mukohataei_DSM_12286:0.02830000,((344211116|Halob|Haloarcula_hispanica
_ATCC_33960:0.00000000,564289120|Halob|Haloarcula_hispanica_N601:0.00000000)0.00:0.00480000
,55377005|Halob|Haloarcula_marismortui_ATCC_43049:0.00522000)0.98:0.03800000)1.00:0.12877000
)0.77:0.01380000)0.93:0.02759000,((222480237|Halob|Halorubrum_lacusprofundi_ATCC_49239:0.094
72000,((292656892|Halob|Haloferax_volcanii_DS2:0.01743000,389848208|Halob|Haloferax_mediterra
nei_ATCC_33500:0.01072000)1.00:0.06867000,(385804346|Halob|Haloquadratum_walsbyi_C23:0.124
51000,313126011|Halob|Halogeometricum_borinquense_DSM_11551:0.03618000)0.87:0.02677000)0.
94:0.03822000)0.44:0.02881000,345004801|uncla|halophilic_archaeon_DL31:0.20560000)0.88:0.0278

6000)0.69:0.02362000,510881120|Halob|Salinarchaeum_sp-_Harcht-
Bsk1:0.12909000)0.44:0.03049000,(452207487|Halob|Natronomonas_moolapensis_8-8-
11:0.10294000,76802065|Halob|Natronomonas_pharaonis_DSM_2160:0.05284000)0.99:0.08513000)0
.83:0.05453000)1.00:0.30242000,(88604126|Metha|Methanospirillum_hungatei_JF-
1:0.39555000,((154151755|Metha|Methanoregula_boonei_6A8:0.08108000,432329926|Metha|Metha
noregula_formicica_SMSP:0.11425000)1.00:0.14906000,((126179752|Metha|Methanoculleus_marisnig
ri_JR1:0.04981000,397779745|Metha|Methanoculleus_bourgensis_MS2:0.07394000)1.00:0.17721000,
(219852951|Metha|Methanosphaerula_palustris_E1-
9c:0.16481000,124486058|Metha|Methanocorpusculum_labreanum_Z:0.29202000)0.96:0.07001000)0
.86:0.02579000)0.98:0.09725000)0.75:0.08472000)0.67:0.07954000)0.09:0.04957000)0.36:0.05118000,
((289596977|uncla|Aciduliprofundum_boonei_T469:0.08189000,432329139|uncla|Aciduliprofundum_
sp-_MAR08-
339:0.09581000)1.00:0.34128000,((13541812|Therm|Thermoplasma_volcanium_GSS1:0.10645000,160
81933|Therm|Thermoplasma_acidophilum_DSM_1728:0.10428000)1.00:0.18749000,(48478306|Ther
m|Picrophilus_torridus_DSM_9790:0.17213000,518652697|Therm|Ferroplasma_acidarmanus_fer1:0.2
1779000)0.99:0.13605000)1.00:0.34309000)1.00:0.21472000)0.52:0.05960000,(((((327311487|Therm|
Thermoproteus_uzoniensis_768-
20:0.07922000,352683060|Therm|Thermoproteus_tenax_Kra_1:0.05432000)1.00:0.13298000,(126458
650|Therm|Pyrobaculum_calidifontis_JCM_11548:0.09279000,((171184538|Therm|Pyrobaculum_neut
rophilum_V24Sta:0.06245000,119872606|Therm|Pyrobaculum_islandicum_DSM_4184:0.07988000)0.8
4:0.02193000,(18312197|Therm|Pyrobaculum_aerophilum_str-
_IM2:0.06532000,(374327727|Therm|Pyrobaculum_sp-
_1860:0.11557000,(145590323|Therm|Pyrobaculum_arsenaticum_DSM_13514:0.01308000,379005396
|Therm|Pyrobaculum_oguniense_TE7:0.00744000)1.00:0.06299000)0.53:0.02505000)0.93:0.03448000)
0.94:0.04341000)1.00:0.13146000)1.00:0.16394000,(159040784|Therm|Caldivirga_maquilingensis_IC-
167:0.37926000,(307594861|Therm|Vulcanisaeta_distributa_DSM_14429:0.03777000,325969095|The
rm|Vulcanisaeta_moutnovskia_768-
28:0.03440000)1.00:0.19466000)0.85:0.08699000)0.98:0.18005000,490715720|uncla|nanoarchaeote_
Nst1:1.26413000)0.94:0.14116000,(557693759|uncla|Candidatus_Caldiarchaeum_subterraneum:0.792
03000,(408403734|Nitro|Candidatus_Nitrososphaera_gargensis_Ga9-
2:0.33414000,(118576791|Cenar|Cenarchaeum_symbiosum_A:0.38035000,((329765802|NULL|NULL:0
.04262000,340344185|NULL|NULL:0.04310000)0.93:0.04477000,(407464178|Nitro|Candidatus_Nitros
opumilus_sp-
_AR2:0.07600000,(161527825|Nitro|Nitrosopumilus_maritimus_SCM1:0.03727000,407461821|Nitro|C
andidatus_Nitrosopumilus_koreensis_AR1:0.00284000)0.98:0.05325000)0.76:0.04842000)1.00:0.17900
000)0.95:0.15360000)1.00:0.51143000)0.98:0.22570000)0.90:0.06590000)0.77:0.04682000)0.84:0.0833
3000)0.98:0.54917000);

TilS/MesJ family

(((((((296109679|Metha|Methanocaldococcus_infernus_ME:0.14183,(261402621|Metha|Methanocal
dococcus_vulcanius_M7:0.12524,(15668662|Metha|Methanocaldococcus_jannaschii_DSM_2661:0.013

46,(256811518|Metha|Methanocaldococcus_fervens_AG86:0.04619,289193222|Metha|Methanocaldo
coccus_sp-_FS406-
22:0.00409)0.074:0.01085)0.914:0.04871)0.831:0.06668)0.974:0.18048,(333910339|Metha|Methanoto
rris_igneus_Kol_5:0.21445,((150401226|Metha|Methanococcus_aeolicus_Nankai-
3:0.18891,336121484|Metha|Methanothermococcus_okinawensis_IH1:0.10079)0.909:0.05649,((15039
8931|Metha|Methanococcus_vannielii_SB:0.12080,45358916|Metha|Methanococcus_maripaludis_S2:
0.09218)0.869:0.06715,(297618556|Metha|Methanococcus_voltae_A3:0.0,297618616|Metha|Methan
ococcus_voltae_A3:0.0):0.31082)0.936:0.07400)0.954:0.10938)0.776:0.07291)0.768:0.40682,(20093491
|Metha|Methanopyrus_kandleri_AV19:0.84915,20094490|Metha|Methanopyrus_kandleri_AV19:1.371
51)0.997:1.05502)0.993:0.84272,((20095126|Metha|Methanopyrus_kandleri_AV19:1.29369,(((3152314
54|Therm|Thermococcus_barophilus_MP:0.13831,(242399643|Therm|Thermococcus_sibiricus_MM_7
39:0.14823,530549058|Therm|Thermococcus_litoralis_DSM_5473:0.06707)0.977:0.05842)0.827:0.027
31,((14521954|Therm|Pyrococcus_abyssi_GE5:0.10387,((18976558|Therm|Pyrococcus_furiosus_DSM_
3638:0.07265,14591705|Therm|Pyrococcus_horikoshii_OT3:0.11922)0.365:0.02930,(332158483|Ther
m|Pyrococcus_sp-_NA2:0.09741,389851782|Therm|Pyrococcus_sp-
_ST04:0.10784)0.904:0.04472)0.780:0.06184)0.991:0.09578,(337285044|Therm|Pyrococcus_yayanosii_
CH1:0.18578,(212224909|Therm|Thermococcus_onnurineus_NA1:0.12404,((341582377|Therm|Therm
ococcus_sp-_4557:0.06170,390961088|Therm|Thermococcus_sp-
_CL1:0.07765)0.795:0.01910,(57641486|Therm|Thermococcus_kodakarensis_KOD1:0.12320,(22347756
0|Therm|Thermococcus_sp-
_AM4:0.06517,240102208|Therm|Thermococcus_gammatolerans_EJ3:0.07790)0.918:0.04171)0.948:0.
03984)0.873:0.02576)0.871:0.06230)0.771:0.05988)0.876:0.03420)0.970:0.30377,(296110048|Metha|
Methanocaldococcus_infernus_ME:0.23622,((333911421|Metha|Methanotorris_igneus_Kol_5:0.17742
,((150401530|Metha|Methanococcus_aeolicus_Nankai-
3:0.25013,336122061|Metha|Methanothermococcus_okinawensis_IH1:0.15703)0.822:0.12335,(29761
9529|Metha|Methanococcus_voltae_A3:0.25511,(150400268|Metha|Methanococcus_vannielii_SB:0.1
2603,45358224|Metha|Methanococcus_maripaludis_S2:0.07944)0.919:0.10240)0.997:0.23334)0.766:0
.12809)1.000:0.23470,(261403285|Metha|Methanocaldococcus_vulcanius_M7:0.10778,(256810280|M
etha|Methanocaldococcus_fervens_AG86:0.07499,(15669795|Metha|Methanocaldococcus_jannaschii
_DSM_2661:0.02488,289191947|Metha|Methanocaldococcus_sp-_FS406-
22:0.05452)0.884:0.03142)0.866:0.04205)0.502:0.03813)0.059:0.09596)0.998:0.46086)0.918:0.42176)0.
998:1.07958,(((530547526|Therm|Thermococcus_litoralis_DSM_5473:0.26649,315230077|Therm|Ther
mococcus_barophilus_MP:0.15353)0.916:0.10405,((223477021|Therm|Thermococcus_sp-
_AM4:0.14985,240103159|Therm|Thermococcus_gammatolerans_EJ3:0.12770)0.939:0.05030,((33728
3994|Therm|Pyrococcus_yayanosii_CH1:0.11378,(332157834|Therm|Pyrococcus_sp-
_NA2:0.15538,((14520772|Therm|Pyrococcus_abyssi_GE5:0.14605,14591383|Therm|Pyrococcus_horik
oshii_OT3:0.17653)0.624:0.06648,(18977994|Therm|Pyrococcus_furiosus_DSM_3638:0.16096,389852
908|Therm|Pyrococcus_sp-
_ST04:0.19049)0.871:0.05619)0.961:0.09222)0.929:0.06429)0.990:0.08141,(57640895|Therm|Thermoc
occus_kodakarensis_KOD1:0.12292,(212223802|Therm|Thermococcus_onnurineus_NA1:0.12440,(3415
81428|Therm|Thermococcus_sp-_4557:0.11394,390960279|Therm|Thermococcus_sp-
_CL1:0.08581)0.480:0.03373)0.934:0.05632)0.695:0.01601)0.597:0.01342)0.518:0.08267)0.949:0.46279
,(20095127|Metha|Methanopyrus_kandleri_AV19:0.94909,(297618698|Metha|Methanococcus_voltae
_A3:0.39860,((333910146|Metha|Methanotorris_igneus_Kol_5:0.10764,(296109523|Metha|Methanoc

aldococcus_infernus_ME:0.30815,((15669205|Metha|Methanocaldococcus_jannaschii_DSM_2661:0.03
211,(256810588|Metha|Methanocaldococcus_fervens_AG86:0.05036,261403540|Metha|Methanocald
ococcus_vulcanius_M7:0.19253)0.986:0.05753)0.551:0.01374,289193095|Metha|Methanocaldococcus
_sp-_FS406-
22:0.02653)0.928:0.07753)1.000:0.24463)0.899:0.06609,((336122045|Metha|Methanothermococcus_
okinawensis_IH1:0.23392,150401671|Metha|Methanococcus_aeolicus_Nankai-
3:0.49123)0.406:0.08361,(150399298|Metha|Methanococcus_vannielii_SB:0.22210,45358795|Metha|
Methanococcus_maripaludis_S2:0.09639)0.999:0.24760)0.782:0.08578)0.900:0.13561)0.991:0.53893)0.
969:0.55220)0.999:1.23075)0.907:0.49184)0.780:0.16223,((((146304252|Therm|Metallosphaera_sedul
a_DSM_5348:0.08487,330834635|Therm|Metallosphaera_cuprina_Ar-
4:0.21439)0.999:0.18645,(332796633|Therm|Acidianus_hospitalis_W1:0.19762,(15920850|Therm|Sulf
olobus_tokodaii_str-
_7:0.17670,70606217|Therm|Sulfolobus_acidocaldarius_DSM_639:0.21032)0.996:0.13618)0.461:0.063
58)1.000:0.45049,(156937500|Therm|Ignicoccus_hospitalis_KIN4-
I:0.82278,347524189|Therm|Pyrolobus_fumarii_1A:1.25326)0.706:0.21417)0.594:0.07656,(((32010061
2|Therm|Desulfurococcus_mucosus_DSM_2162:0.38294,(218884033|Therm|Desulfurococcus_kamcha
tkensis_1221n:0.02506,390938516|Therm|Desulfurococcus_fermentans_DSM_16532:0.04128)0.980:0.
16872)1.000:0.54590,((126465307|Therm|Staphylothermus_marinus_F1:0.08973,297526412|Therm|S
taphylothermus_hellenicus_DSM_12710:0.05997)1.000:0.47265,((307596367|Therm|Vulcanisaeta_dist
ributa_DSM_14429:0.50828,(119720174|Therm|Thermofilum_pendens_Hrk_5:0.61705,(124026994|Th
erm|Hyperthermus_butylicus_DSM_5456:0.47670,(14601833|Therm|Aeropyrum_pernix_K1:0.03658,5
49455949|Therm|Aeropyrum_camini_SY1_-
_JCM_12091:0.05017)1.000:0.38145)0.892:0.09871)0.875:0.10350)0.639:0.07869,(302348716|Therm|
Acidilobus_saccharovorans_345-
15:0.74665,(389861716|Therm|Thermogladius_cellulolyticus_1633:0.60752,305662983|Therm|Ignisph
aera_aggregans_DSM_17230:0.57401)0.714:0.17196)0.057:0.06453)0.222:0.05079)0.042:0.05701)0.99
0:0.18172,(((41615074|Nanoa|Nanoarchaeum_equitans_Kin4-
M:0.94412,170289837|Candi|Candidatus_Korarchaeum_cryptofilum_OPF8:1.26050)0.792:0.17555,(((1
26460621|Therm|Pyrobaculum_calidifontis_JCM_11548:0.06181,(119871537|Therm|Pyrobaculum_isl
andicum_DSM_4184:0.12742,(18314221|Therm|Pyrobaculum_aerophilum_str-
_IM2:0.10632,379004198|Therm|Pyrobaculum_oguniense_TE7:0.13618)0.993:0.10135)0.963:0.13198)
1.000:0.94790,(289596443|uncla|Aciduliprofundum_boonei_T469:0.10085,432328216|uncla|Acidulipr
ofundum_sp-_MAR08-
339:0.16754)1.000:0.63082)0.939:0.24953,(288931458|Archa|Ferroglobus_placidus_DSM_10642:0.367
99,(327401576|Archa|Archaeoglobus_veneficus_SNP6:0.34644,(284161871|Archa|Archaeoglobus_pro
fundus_DSM_5631:0.36313,11498919|Archa|Archaeoglobus_fulgidus_DSM_4304:0.24273)0.895:0.157
31)0.937:0.11858)0.971:0.17538)0.918:0.11308)0.890:0.08950,((333911645|Metha|Methanotorris_ign
eus_Kol_5:0.29776,(296108939|Metha|Methanocaldococcus_infernus_ME:0.16409,(261403374|Meth
a|Methanocaldococcus_vulcanius_M7:0.12532,(15669670|Metha|Methanocaldococcus_jannaschii_DS
M_2661:0.03087,(256811396|Metha|Methanocaldococcus_fervens_AG86:0.07404,289192452|Metha|
Methanocaldococcus_sp-_FS406-
22:0.03383)0.056:0.01012)0.950:0.04934)0.988:0.11738)1.000:0.28390)0.873:0.09246,(((337284842|Th
erm|Pyrococcus_yayanosii_CH1:0.08882,(18976645|Therm|Pyrococcus_furiosus_DSM_3638:0.09782,((
389851871|Therm|Pyrococcus_sp-_ST04:0.08110,(332158560|Therm|Pyrococcus_sp-

_NA2:0.08484,14590222|Therm|Pyrococcus_horikoshii_OT3:0.05337)0.826:0.02107)0.981:0.04559,145
21864|Therm|Pyrococcus_abyssi_GE5:0.02976)0.867:0.02308)0.796:0.03561)0.959:0.08654,((5764149
1|Therm|Thermococcus_kodakarensis_KOD1:0.05579,(240104187|Therm|Thermococcus_gammatoler
ans_EJ3:0.02294,(((212224868|Therm|Thermococcus_onnurineus_NA1:0.03348,341582427|Therm|Th
ermococcus_sp-_4557:0.04691)0.904:0.02026,390961133|Therm|Thermococcus_sp-
_CL1:0.03204)0.957:0.02943,223477951|Therm|Thermococcus_sp-
_AM4:0.01364)0.142:0.01775)0.903:0.03383)0.982:0.06201,(315231707|Therm|Thermococcus_baroph
ilus_MP:0.11924,(242398445|Therm|Thermococcus_sibiricus_MM_739:0.11262,530547921|Therm|Th
ermococcus_litoralis_DSM_5473:0.06723)0.955:0.05099)0.570:0.03187)0.916:0.04830)0.927:0.07652,3
12137387|Metha|Methanothermus_fervidus_DSM_2088:0.48386)0.747:0.07526)0.999:0.28968)0.943:
0.12023)0.822:0.07109)0.999:0.29721)0.967:0.15724,((557694617|uncla|Candidatus_Caldiarchaeum_s
ubterraneum:0.74505,(408403438|Nitro|Candidatus_Nitrososphaera_gargensis_Ga9-
2:0.30605,(118575266|Cenar|Cenarchaeum_symbiosum_A:0.22917,(407463919|Nitro|Candidatus_Nit
rosopumilus_sp-
_AR2:0.07296,((161527617|Nitro|Nitrosopumilus_maritimus_SCM1:0.03742,407461606|Nitro|Candida
tus_Nitrosopumilus_koreensis_AR1:0.02155)0.962:0.03465,(329766712|NULL|NULL:0.03453,34034397
1|NULL|NULL:0.03931)0.999:0.06854)0.556:0.02895)0.998:0.21563)0.977:0.17770)1.000:0.44151)0.71
6:0.12641,((159041820|Therm|Caldivirga_maquilingensis_IC-
167:0.28096,(307594196|Therm|Vulcanisaeta_distributa_DSM_14429:0.04087,325968454|Therm|Vulc
anisaeta_moutnovskia_768-
28:0.08028)0.941:0.07691)0.760:0.15149,(((((374326060|Therm|Pyrobaculum_sp-
_1860:0.10065,18313243|Therm|Pyrobaculum_aerophilum_str-
_IM2:0.08961)0.917:0.02824,(171185906|Therm|Pyrobaculum_neutrophilum_V24Sta:0.09603,119872
527|Therm|Pyrobaculum_islandicum_DSM_4184:0.08553)0.873:0.04145)0.656:0.02401,(145591717|T
herm|Pyrobaculum_arsenaticum_DSM_13514:0.00603,379003663|Therm|Pyrobaculum_oguniense_TE
7:0.00513)1.000:0.09166)0.973:0.06219,126460252|Therm|Pyrobaculum_calidifontis_JCM_11548:0.05
949)0.994:0.10952,(327310455|Therm|Thermoproteus_uzoniensis_768-
20:0.14715,352682139|Therm|Thermoproteus_tenax_Kra_1:0.15759)0.966:0.09171)0.982:0.16181)1.0
00:0.61049)0.899:0.15094)0.444:0.07641,((156937300|Therm|Ignicoccus_hospitalis_KIN4-
I:0.67461,(385805212|Therm|Fervidicoccus_fontis_Kam940:0.44087,(((146304686|Therm|Metallospha
era_sedula_DSM_5348:0.16492,330834210|Therm|Metallosphaera_cuprina_Ar-
4:0.13161)0.988:0.13354,(332796837|Therm|Acidianus_hospitalis_W1:0.14147,((15897505|Therm|Sul
folobus_solfataricus_P2:0.04716,238619984|Therm|Sulfolobus_islandicus_M-16-
4:0.03747)1.000:0.19710,(70607306|Therm|Sulfolobus_acidocaldarius_DSM_639:0.25718,15921747|T
herm|Sulfolobus_tokodaii_str-
_7:0.04390)0.991:0.10382)0.148:0.03895)0.626:0.04843)1.000:0.32145,(305662530|Therm|Ignisphaer
a_aggregans_DSM_17230:0.54492,((118431097|Therm|Aeropyrum_pernix_K1:0.02718,549455057|Th
erm|Aeropyrum_camini_SY1_-
_JCM_12091:0.02796)1.000:0.41876,(302348148|Therm|Acidilobus_saccharovorans_345-
15:0.16417,429216921|Therm|Caldisphaera_lagunensis_DSM_15908:0.33098)0.988:0.21045)0.536:0.1
4017)0.922:0.13971)0.718:0.11439)0.973:0.19305)0.981:0.21183,(170290828|Candi|Candidatus_Korar
chaeum_cryptofilum_OPF8:0.99720,20094539|Metha|Methanopyrus_kandleri_AV19:0.55099)0.837:0.
21947)0.061:0.10088)0.495:0.03235,(((510882260|Halob|Salinarchaeum_sp-_Harcht-
Bsk1:0.24666,((((452208566|Halob|Natronomonas_moolapensis_8-8-

11:0.06190,76803288|Halob|Natronomonas_pharaonis_DSM_2160:0.07690)1.000:0.20562,((15789499
|Halob|Halobacterium_sp-_NRC-
1:0.0,169235215|Halob|Halobacterium_salinarum_R1:0.0):0.26958,(257053471|Halob|Halorhabdus_u
tahensis_DSM_12940:0.17995,(257386246|Halob|Halomicrobium_mukohataei_DSM_12286:0.07058,((
344212765|Halob|Haloarcula_hispanica_ATCC_33960:0.0,564290696|Halob|Haloarcula_hispanica_N6
01:0.0):0.00363,55378723|Halob|Haloarcula_marismortui_ATCC_43049:0.00349)0.998:0.08396)0.931:
0.05680)0.849:0.04594)0.481:0.03538)0.852:0.02708,(300711905|Halob|Halalkalicoccus_jeotgali_B3:0.
12778,(336253642|Halob|Halopiger_xanaduensis_SH-6:0.03852,((((397773902|Halob|Natrinema_sp-
_J7-
2:0.02920,435847210|Halob|Natronococcus_occultus_SP4:0.06229)0.948:0.02870,(433590134|Halob|
Natrinema_pellirubrum_DSM_15624:0.06355,284166087|Halob|Haloterrigena_turkmenica_DSM_5511
:0.02840)0.142:0.00572)0.840:0.01439,433639340|Halob|Halovivax_ruber_XH-
70:0.13297)0.780:0.01272,(429192440|Halob|Natronobacterium_gregoryi_SP2:0.07408,289580657|Ha
lob|Natrialba_magadii_ATCC_43099:0.05285)0.334:0.01294)0.894:0.02363)0.997:0.08067)0.949:0.0531
3)0.149:0.01863,((345006050|uncla|halophilic_archaeon_DL31:0.14302,222478949|Halob|Halorubru
m_lacusprofundi_ATCC_49239:0.18571)0.151:0.02408,(313127354|Halob|Halogeometricum_borinque
nse_DSM_11551:0.13077,(292654746|Halob|Haloferax_volcanii_DS2:0.00252,389846032|Halob|Halof
erax_mediterranei_ATCC_33500:0.01550)0.999:0.09442)0.906:0.03304)0.898:0.03221)0.749:0.04462)1.
000:0.62549,(((296109212|Metha|Methanocaldococcus_infernus_ME:0.39866,(261403320|Metha|Me
thanocaldococcus_vulcanius_M7:0.21951,(256810699|Metha|Methanocaldococcus_fervens_AG86:0.0
6431,(15669347|Metha|Methanocaldococcus_jannaschii_DSM_2661:0.05035,289192268|Metha|Met
hanocaldococcus_sp-_FS406-
22:0.04887)0.959:0.07643)0.966:0.09466)0.315:0.05062)0.972:0.23312,(333910340|Metha|Methanoto
rris_igneus_Kol_5:0.14838,(((150398925|Metha|Methanococcus_vannielii_SB:0.0,150399417|Metha|
Methanococcus_vannielii_SB:0.0):0.15789,(297619559|Metha|Methanococcus_voltae_A3:0.24107,453
58919|Metha|Methanococcus_maripaludis_S2:0.14781)0.539:0.08109)0.971:0.09537,(150401224|Met
ha|Methanococcus_aeolicus_Nankai-
3:0.28541,336121482|Metha|Methanothermococcus_okinawensis_IH1:0.10517)0.913:0.04943)1.000:0
.19961)0.000:0.09847)1.000:0.51136,((385806039|Therm|Fervidicoccus_fontis_Kam940:2.27510,(3056
63585|Therm|Ignisphaera_aggregans_DSM_17230:2.11188,(429217398|Therm|Caldisphaera_lagunen
sis_DSM_15908:0.77847,(118431597|Therm|Aeropyrum_pernix_K1:0.13605,549455772|Therm|Aerop
yrum_camini_SY1_-
_JCM_12091:0.14626)1.000:1.52571)0.878:0.42143)0.746:0.17801)0.994:1.12026,330506961|Metha|
Methanosaeta_concilii_GP6:1.10780)0.906:0.28638)0.802:0.06598)0.721:0.02049,(312136595|Metha|
Methanothermus_fervidus_DSM_2088:0.33879,(84489152|Metha|Methanosphaera_stadtmanae_DSM
_3091:0.35458,((148642613|Metha|Methanobrevibacter_smithii_ATCC_35061:0.24956,(288559819|M
etha|Methanobrevibacter_ruminantium_M1:0.10513,509155503|Metha|Methanobrevibacter_sp-
_AbM4:0.22904)0.867:0.08742)0.985:0.13159,((15679734|Metha|Methanothermobacter_thermautotr
ophicus_str-_Delta_H:0.03339,304314090|Metha|Methanothermobacter_marburgensis_str-
_Marburg:0.09540)1.000:0.24711,(333988106|Metha|Methanobacterium_paludis:0.14382,(325958250
|Metha|Methanobacterium_lacus:0.17962,566003536|Metha|Methanobacterium_sp-
_MB1:0.18732)0.315:0.04029)0.811:0.06168)0.292:0.05940)0.845:0.06183)0.964:0.12283)1.000:0.3015
2)0.192:0.04152,((((566003702|Metha|Methanobacterium_sp-
_MB1:0.32637,(((325958096|Metha|Methanobacterium_lacus:0.31608,333988276|Metha|Methanoba

cterium_paludis:0.17688)0.751:0.09160,(((15679197|Metha|Methanothermobacter_thermautotrophic
us_str-_Delta_H:0.11269,304315308|Metha|Methanothermobacter_marburgensis_str-
_Marburg:0.17244)1.000:0.32176,(509155614|Metha|Methanobrevibacter_sp-
_AbM4:0.14392,(148643088|Metha|Methanobrevibacter_smithii_ATCC_35061:0.18391,288561212|M
etha|Methanobrevibacter_ruminantium_M1:0.13258)0.937:0.06995)0.994:0.17746)0.741:0.06858,312
137453|Metha|Methanothermus_fervidus_DSM_2088:0.58553)0.901:0.07864)0.113:0.03316,8448911
0|Metha|Methanosphaera_stadtmanae_DSM_3091:0.71338)0.289:0.07507)0.999:0.45495,((51105963
1|Therm|Candidatus_Methanomassiliicoccus_intestinalis:0.37323,(474934290|Therm|Thermoplasmat
ales_archaeon_BRNA1:0.13985,478483048|uncla|Candidatus_Methanomethylophilus_alvus_Mx1201:0
.07359)1.000:0.36467)0.991:0.18021,((289596819|uncla|Aciduliprofundum_boonei_T469:0.12091,432
329346|uncla|Aciduliprofundum_sp-_MAR08-
339:0.07315)1.000:0.40537,((13541276|Therm|Thermoplasma_volcanium_GSS1:0.14621,16082141|Th
erm|Thermoplasma_acidophilum_DSM_1728:0.18111)0.999:0.20861,(48477224|Therm|Picrophilus_to
rridus_DSM_9790:0.29353,518651809|Therm|Ferroplasma_acidarmanus_fer1:0.16158)0.987:0.13643)
0.999:0.27834)0.907:0.08328)0.996:0.17387)0.102:0.03785,(((119719832|Therm|Thermofilum_penden
s_Hrk_5:0.15565,530780780|Therm|Thermofilum_sp-
_1910b:0.13548)1.000:0.68710,((389861274|Therm|Thermogladius_cellulolyticus_1633:0.22268,(((126
464914|Therm|Staphylothermus_marinus_F1:0.04215,297526784|Therm|Staphylothermus_hellenicus
_DSM_12710:0.01031)0.996:0.15315,(124027476|Therm|Hyperthermus_butylicus_DSM_5456:0.06485
,347524311|Therm|Pyrolobus_fumarii_1A:0.10741)1.000:0.22521)0.975:0.12432,(296242202|Therm|T
hermosphaera_aggregans_DSM_11486:0.22313,(320100819|Therm|Desulfurococcus_mucosus_DSM_2
162:0.11089,(218883803|Therm|Desulfurococcus_kamchatkensis_1221n:0.01239,390938185|Therm|
Desulfurococcus_fermentans_DSM_16532:0.01332)0.999:0.13962)0.997:0.15708)0.918:0.09463)0.757:
0.06362)0.995:0.27952,(490715585|uncla|nanoarchaeote_Nst1:0.59721,41615304|Nanoa|Nanoarcha
eum_equitans_Kin4-
M:0.43047)0.999:0.38082)0.287:0.12972)0.902:0.13491,((315231685|Therm|Thermococcus_barophilu
s_MP:0.02903,((242399147|Therm|Thermococcus_sibiricus_MM_739:0.03873,530547516|Therm|Ther
mococcus_litoralis_DSM_5473:0.01821)0.997:0.06061,(((57641756|Therm|Thermococcus_kodakarensi
s_KOD1:0.02302,(240102078|Therm|Thermococcus_gammatolerans_EJ3:0.00748,223477482|Therm|T
hermococcus_sp-_AM4:0.00374)0.864:0.00766)0.857:0.00767,(390961162|Therm|Thermococcus_sp-
_CL1:0.01727,212224843|Therm|Thermococcus_onnurineus_NA1:0.03825)0.903:0.01375)0.549:0.0116
3,341582453|Therm|Thermococcus_sp-
_4557:0.03127)0.996:0.07811)0.758:0.02179)0.863:0.06241,(337284013|Therm|Pyrococcus_yayanosii_
CH1:0.08731,(18978130|Therm|Pyrococcus_furiosus_DSM_3638:0.04819,(332157910|Therm|Pyrococ
cus_sp-
_NA2:0.00364,(14520709|Therm|Pyrococcus_abyssi_GE5:0.00761,14591444|Therm|Pyrococcus_horik
oshii_OT3:0.01514)0.608:0.00396)0.849:0.01434)0.194:0.03122)0.840:0.03374)0.996:0.21000)0.990:0.1
5516)0.478:0.03295,(((((336477052|Metha|Methanosalsum_zhilinae_DSM_4017:0.22738,(((73670143|
Metha|Methanosarcina_barkeri_str-
_Fusaro:0.03723,(20090822|Metha|Methanosarcina_acetivorans_C2A:0.01743,(21228898|Metha|Met
hanosarcina_mazei_Go1:0.0,452211286|Metha|Methanosarcina_mazei_Tuc01:0.0):0.03909)0.976:0.06
578)1.000:0.14167,(294496034|Metha|Methanohalophilus_mahii_DSM_5219:0.20433,91772989|Met
ha|Methanococcoides_burtonii_DSM_6242:0.15823)0.922:0.08676)0.763:0.03018,((410669608|Metha
|Methanolobus_psychrophilus_R15:0.14345,435851183|Metha|Methanomethylovorans_hollandica_D

SM_15978:0.19883)0.959:0.07442,298674631|Metha|Methanohalobium_evestigatum_Z-
7303:0.24761)0.459:0.03758)0.841:0.07601)0.994:0.16781,(116753888|Metha|Methanosaeta_thermo
phila_PT:0.25860,386001198|Metha|Methanosaeta_harundinacea_6Ac:0.41075)0.999:0.24887)0.365:0
.04554,((147921431|Metha|Methanocella_arvoryzae_MRE50:0.11516,(282164695|Metha|Methanocel
la_paludicola_SANAE:0.09665,383320454|Metha|Methanocella_conradii_HZ254:0.12075)0.721:0.0655
9)1.000:0.30675,(307354722|Metha|Methanoplanus_petrolearius_DSM_11571:0.35132,(154151861|
Metha|Methanoregula_boonei_6A8:0.94061,397779611|Metha|Methanoculleus_bourgensis_MS2:0.1
7972)0.731:0.13654)1.000:0.33034)0.782:0.05161)0.817:0.03275,(330508540|Metha|Methanosaeta_c
oncilii_GP6:0.47502,((219851846|Metha|Methanosphaerula_palustris_E1-
9c:0.50154,124485791|Metha|Methanocorpusculum_labreanum_Z:0.49672)0.898:0.15025,((15415166
0|Metha|Methanoregula_boonei_6A8:0.37534,432330227|Metha|Methanoregula_formicica_SMSP:0.
30180)0.895:0.16570,88603458|Metha|Methanospirillum_hungatei_JF-
1:0.94706)0.831:0.09356)0.998:0.27510)0.874:0.12130)0.973:0.10042,(288932186|Archa|Ferroglobus_
placidus_DSM_10642:0.23854,(((11499188|Archa|Archaeoglobus_fulgidus_DSM_4304:0.11653,284162
492|Archa|Archaeoglobus_profundus_DSM_5631:0.17842)0.757:0.05385,327401180|Archa|Archaeogl
obus_veneficus_SNP6:0.10691)0.286:0.04281,488601045|Archa|Archaeoglobus_sulfaticallidus_PM70-
1:0.17151)0.914:0.07399)1.000:0.27400)0.499:0.04939)0.847:0.07635);
